# Effects of non-invasive trigeminal nerve stimulation frequency and duration on cardiovascular responses

**DOI:** 10.1007/s13534-025-00518-z

**Published:** 2025-11-03

**Authors:** Chaeyeon Kim, Seokbeen Lim, Hun-gyeom Kim, Dohyoung Kim, Youngmin Park, Joowan Seo, Dong Pyo Jang

**Affiliations:** 1 Department of Electronic Engineering, Hanyang University, Seoul, Republic of Korea; 2 Department of Radiology, Mayo Clinic, Rochester, MN USA; 3 Department of Biomedical Engineering, Hanyang University, Seoul, Republic of Korea; 4 Nu Eyne Co., Ltd., Seoul, Korea

**Keywords:** Trigeminal nerve, Electrical stimulation, Cardiovascular response, Autonomic nervous system

## Abstract

This study investigated the effects of trigeminal nerve (TN) stimulation on cardiovascular responses in healthy individuals. Sixty-one participants received electrical stimulation to the ophthalmic and maxillary branches of the trigeminal nerve at different frequencies (2 Hz, 20 Hz, and 200 Hz) while heart rate (HR), pulse arrival time (PAT), and heart rate variability (HRV) were monitored. Results demonstrated frequency-dependent cardiovascular responses, with higher frequencies (particularly 200 Hz) producing more pronounced effects on both HR and PAT. HR showed significant decreases during stimulation, with recovery times proportional to stimulation frequency. PAT changes, which inversely reflect blood pressure alterations, occurred more rapidly than HR changes, suggesting baroreflex-mediated regulation. Notably, habituation effects were observed with repeated stimulation at 2-min intervals, but these effects were minimized when using shorter (30-s) stimulation periods. HRV analysis revealed a significant negative correlation between resting LF/HF ratio and stimulation-induced changes, indicating that TN stimulation particularly influences autonomic balance in individuals with sympathetic hyperactivity. These findings provide insights into the mechanisms of TN stimulation on cardiovascular function and suggest potential therapeutic applications for conditions characterized by autonomic dysregulation.

## Introduction

Trigeminal nerve (TN) stimulation has emerged as a promising neuromodulation technique for treating various psychiatric and neurological disorders [1–6]. Studies have shown that TN stimulation is effective in treating epilepsy [7], with patients who received stimulation to the ophthalmic branch of the TN for up to 12 months experiencing more than a 50% reduction in seizure frequency after just 3 months of treatment. Additionally, Shiozawa and colleagues demonstrated that stimulating the orbital branch of the TN for 30 min significantly reduced depressive symptoms in patients with major depressive disorder [8]. Furthermore, a recent systematic review and meta-analysis by Westwood, S. J. et al. revealed substantial improvements in both depression symptoms and quality of life in patients receiving external TN stimulation, especially when used in conjunction with anti-migraine medication. Their analysis also highlighted the technique’s excellent tolerability and strong safety profile [9].

While these therapeutic applications of TN stimulation have shown promising results in neurological and psychiatric disorders, researchers have also begun to investigate its effects on other physiological systems, particularly its influence on cardiovascular function through what is known as the trigemino-cardiac reflex (TCR) [10–13]. This relationship between TN stimulation and cardiovascular function, especially heart rate (HR), has become an important area of scientific inquiry. The TCR typically involves a decrease in both HR and blood pressure (BP) when the TN is stimulated [14], which has significant implications for various medical procedures and treatments. Key studies have highlighted the significant impact of TN stimulation on cardiovascular parameters [15]. For instance, Lapi et al. explored the effects of TN proprioceptive stimulation by mandibular extension on rat BP, HR, and pial microcirculation, finding that TN stimulation significantly decreased mean arterial BP and HR, indicating specific regulatory mechanisms in systemic arterial pressure and cerebral hemodynamics [16]. Additionally, Duan et al. conducted a study on TCR during skull base surgery, analyzing 291 patients. The study identified a significant drop-in HR and mean arterial BP coinciding with TN manipulation, emphasizing the importance of recognizing and managing TCR during such procedures [17].

Electroceuticals such as TN stimulation, which involve the use of electrical stimulation for therapeutic purposes, have shown promising potential in treating various conditions. However, studies specifically examining the relationship between TN stimulation and heart function in humans are relatively scarce. This gap in research is significant because changes in HR in response to TN stimulation may indicate the treatment’s potential efficacy. For instance, Liu et al. investigated the predictive value of preoperative heart rate variability (HRV) for vagus nerve stimulation (VNS) outcomes in patients with drug-resistant epilepsy [18]. Their findings suggested that HRV assessments could help predict the efficacy of neuromodulation treatments, including TN stimulation, highlighting the importance of cardiovascular responses as potential markers for the success of TN stimulation therapies. This underscores the need for more human studies to explore the cardiovascular effects of TN stimulation and its therapeutic implications fully.

In this study, we hypothesized that TN stimulation at higher frequencies would induce greater modulation of cardiovascular function and that shorter stimulation durations would mitigate habituation. Thus, we examined cardiovascular responses to TN stimulation using various frequencies (2 Hz, 20 Hz, and 200 Hz) delivered at 2-min intervals, measuring both HR and pulse arrival time (PAT) changes against a sham condition. To investigate adaptation effects, researchers conducted two protocols: three consecutive 2-min stimulations and ten consecutive 30-s stimulations. In addition, HRV analysis was performed to evaluate parasympathetic influence, specifically measuring the LF/HF ratio at rest and during stimulation to determine correlation patterns between baseline autonomic balance and stimulation-induced changes.

## Methods

### Subject

Healthy adults without any known cardiovascular or neurological disorders, voluntarily recruited through public advertisements. Sixty-one healthy participants (30 males and 31 females, mean age: 22.21 ± 3.15 years) were enrolled in the study. The sample size for each group was determined with reference to previous neuromodulation studies using TN stimulation [19, 20]. Sixteen participants were assigned to the sham group, and 15 participants each to the 2, 20, and 200-Hz stimulation groups, yielding a total sample of 61.

**Fig. 1:**
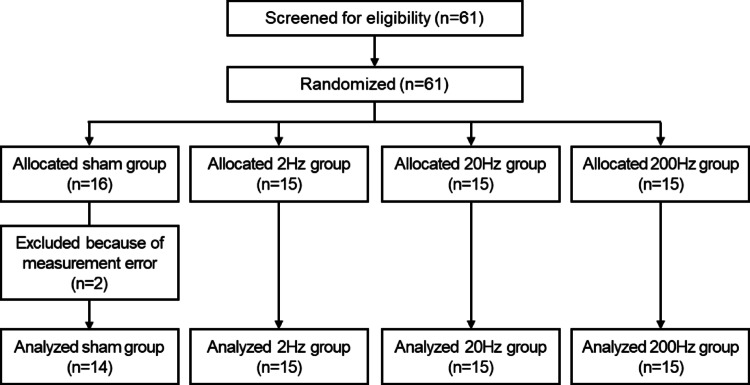
Flow diagram of participant recruitment and inclusion in analysis

Participants were randomly assigned to one of the stimulation frequency groups (sham, 2 Hz, 20 Hz, and 200 Hz) in a single-blind design, such that they were unaware of their group allocation. Two participants in the sham group were excluded from the final analysis due to data loss caused by measurement equipment errors. The flow diagram illustrating participant recruitment and inclusion in the final analysis is presented in Fig. 1, and the demographic characteristics of each group are summarized in Table 1.

**Table 1:** Participant characteristics

Group	Sham	2 Hz	20 Hz	200 Hz
The number of subjects	16	15	15	15
Age (Mean ± SD)	22.19 ± 3.36	23.33 ± 4.51	22.07 ± 1.56	21.27 ± 1.57
Gender (Female)	7 (9)	9 (6)	8 (7)	8 (7)
BMI (Mean ± SD)	21.6 ± 3.08	23.22 ± 2.75	24.22 ± 2.66	21.52 ± 2.76

Participants were instructed to abstain from drinking alcohol, smoking, and consuming caffeine the day prior to the experiment. Individuals with cardiovascular disease were excluded from participation. The experimental procedures were reviewed and approved by the Institutional Review Board of Hanyang University (Approval No. HYU-2020-03-010). All participants provided written informed consent and received a small financial incentive for their participation.

### Experiment protocol

**Fig. 2:**
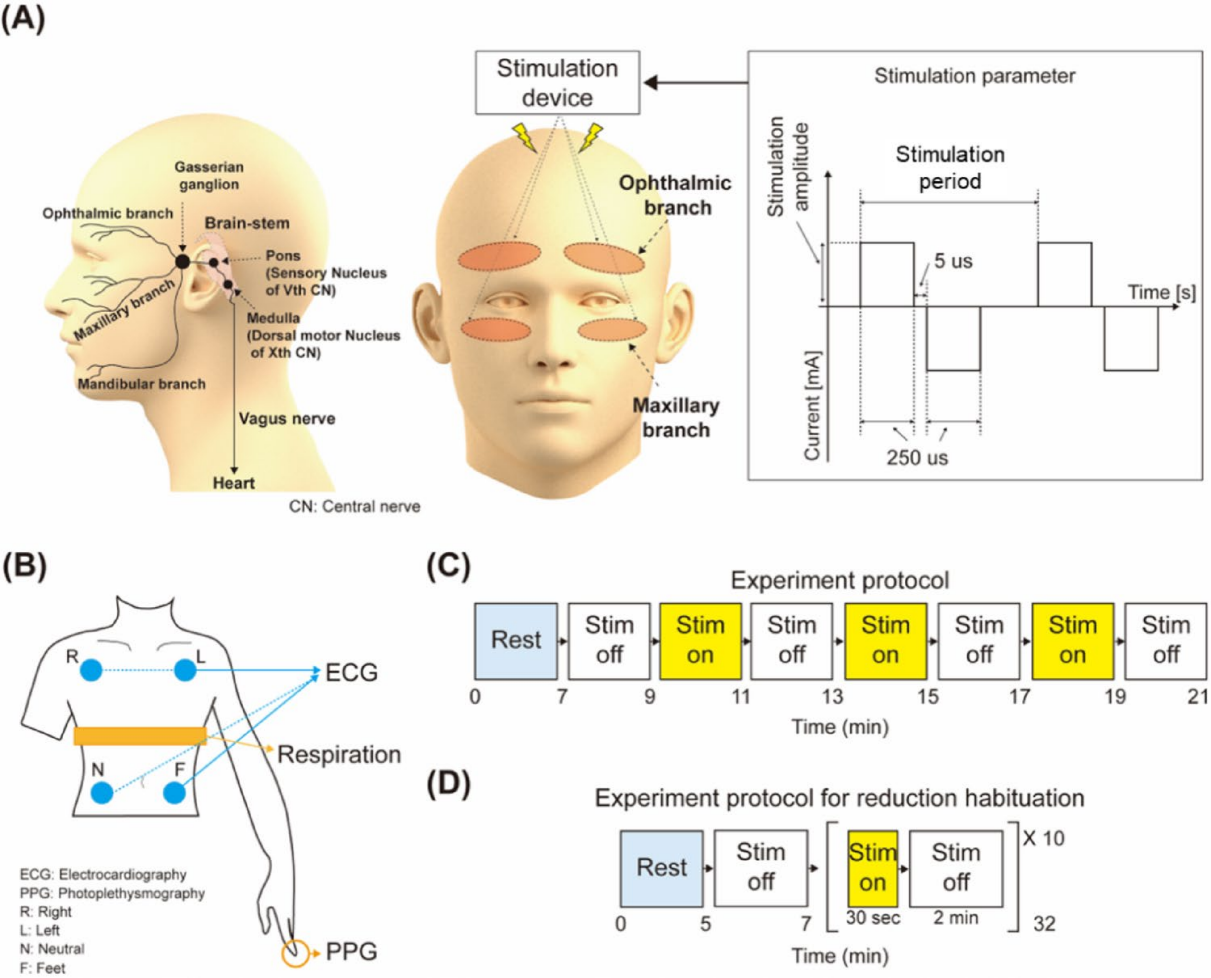
Overview of experimental setup and data acquisition. **A** The stimulation sites and parameters. The stimulation was applied to the ophthalmic and maxillary branches of the TN on the face. **B** The locations for bio-signal data acquisition, where blue indicates ECG measurement sites, yellow represents respiration monitoring sites, and orange denotes PPG measurement locations. **C** The experimental protocol, with the blue lines indicating rest periods and the yellow lines representing stimulation periods

The experiments were conducted in the late morning (between 9:00 AM and 12:00 PM). Before the experiment began, participants were attached with Ag/AgCl electrodes (Kendall 100 series foam electrodes, Cardinal Health, Inc., USA) for electrocardiography (ECG) measurement at four locations on the chest and abdomen (Fig. 2B). Photoplethysmography (PPG) sensor was attached to the tip of the finger. To minimize changes in autonomic nervous system activity, participants were instructed to maintain a respiratory rate of 20 breaths per minute, synchronized with a metronome (FM-310, Fzone) and the respiration signal was monitored by band-type respiration sensor (TSD201, Biopac System Inc, USA). To reduce extraneous noise, participants wore wireless noise-canceling earphones (AirPods Pro, Apple).

The experimental protocol, as depicted in Fig. 2C, lasted 21 min. It began with a 7-min resting baseline, followed by alternating 2-min sections with electrical stimulation OFF and ON, repeated three times. The protocol concluded with a final 2-min OFF period. During the experiment, ECG, PPG, and respiration signals were continuously recorded in real time. ECG and PPG signals were sampled at 1000 Hz using the Taskforce Monitor (Taskforce Monitor 3040i, Austria), whereas respiration signals were sampled at 2000 Hz using the Biopac system (MP160, Biopac Systems Inc., USA). This setup was necessary because the Taskforce Monitor does not include a respiration measurement module; thus, a separate Biopac system was employed for monitoring respiration. The Biopac system is configured to acquire high-resolution signals by default, which resulted in the respiration signal being recorded at a higher sampling frequency. In this study, respiration signals were used to confirm that participants were following the instructed breathing protocol; therefore, they were not included in the analysis or presented in the results.

### Electrical stimulation setting

Medical low-frequency stimulation pads (Nu Eyne Co., Ltd., Korea) were placed on the forehead, just above the eyebrows, and on the cheeks, just below the eyes. These pads were specifically designed to target the ophthalmic and maxillary branches of the TN. Electrical stimulation was delivered using Nu Eyne’s clinical trial stimulator (TPD-NH1, Nu Eyne Co., Ltd., Korea) in biphasic pulses with a phase duration of 250 µs. The stimulation frequencies were set to 2 Hz, 20 Hz, and 200 Hz, depending on the group (Fig. 2A). Participants adjusted the stimulation intensity themselves to below level where they experienced mild discomfort. The average stimulation intensities were as follows: 2 Hz group: 3.39 ± 1.11 mA, 20 Hz group: 2.99 ± 1.11 mA, and 200 Hz group: 2.60 ± 0.80 mA.

### Data analysis

All bio-signal data were processed using MATLAB software (MathWorks, USA). First, the R–R interval (RRI) was determined by identifying the R peaks in the ECG signal. Continuous HR was then calculated by dividing each RRI into 60 s after resampling. The calculated HR values for each group were averaged and smoothed using a moving median filter with a window length of 5 s.

Feature points from the PPG signal and the RRI from the ECG were extracted to calculate the PAT, defined as the time difference between the R peak of the ECG and the corresponding feature point in the PPG. The feature point in the PPG was identified as the maximum peak of the first derivative of the PPG signal. After resampling, the PAT values were averaged and filtered with a moving median filter using a 5-s window.

To evaluate changes in cardiovascular signals between stimulation ON and OFF phases, a normal probability density distribution was constructed using the mean and standard deviation of the preceding OFF phase. This distribution was then used as a reference to quantify how much each data point in the ON phase deviated from the baseline variability. A two-tailed test with a significance level of 0.05 was applied, and Bonferroni correction was implemented to adjust for multiple comparisons. This analytical procedure was applied consistently to both HR and PAT datasets. All statistical analyses were performed using functions from MATLAB’s Statistics and Machine Learning Toolbox.

Autonomic nervous system (ANS) indicators were analyzed using the HRVAS toolbox, a MATLAB add-on. HRV analysis was conducted across all TN stimulation groups (2, 20, and 200 Hz; sham group was excluded; N = 45). The high-frequency (HF, 0.15–0.40 Hz) band of HRV was used as a marker of parasympathetic activity, while the low-frequency (LF, 0.04–0.15 Hz) band represented sympathetic activity. To account for the fixed respiratory rate during the experiment (20 breaths per minute, equivalent to 0.33 Hz), the HF band was adjusted to 0.15–0.3 Hz. ANS balance was quantified using the LF/HF ratio, which reflects the relative contributions of sympathetic and parasympathetic activity. To further evaluate the effects of TN stimulation on autonomic balance, the correlation analysis was performed to examine the relationship between the LF/HF ratio in the rest phase and the change in LF/HF ratio during the first stimulation ON phase. Prior to this analysis, the normality of both variables was assessed using the Shapiro–Wilk test, which indicated that neither variable followed a normal distribution (*p* < 0.05). Consequently, Spearman’s rank correlation, a non-parametric method, was applied to assess the association between these variables.

## Results

### Frequency-dependent cardiovascular responses to trigeminal nerve stimulation

**Fig. 3:**
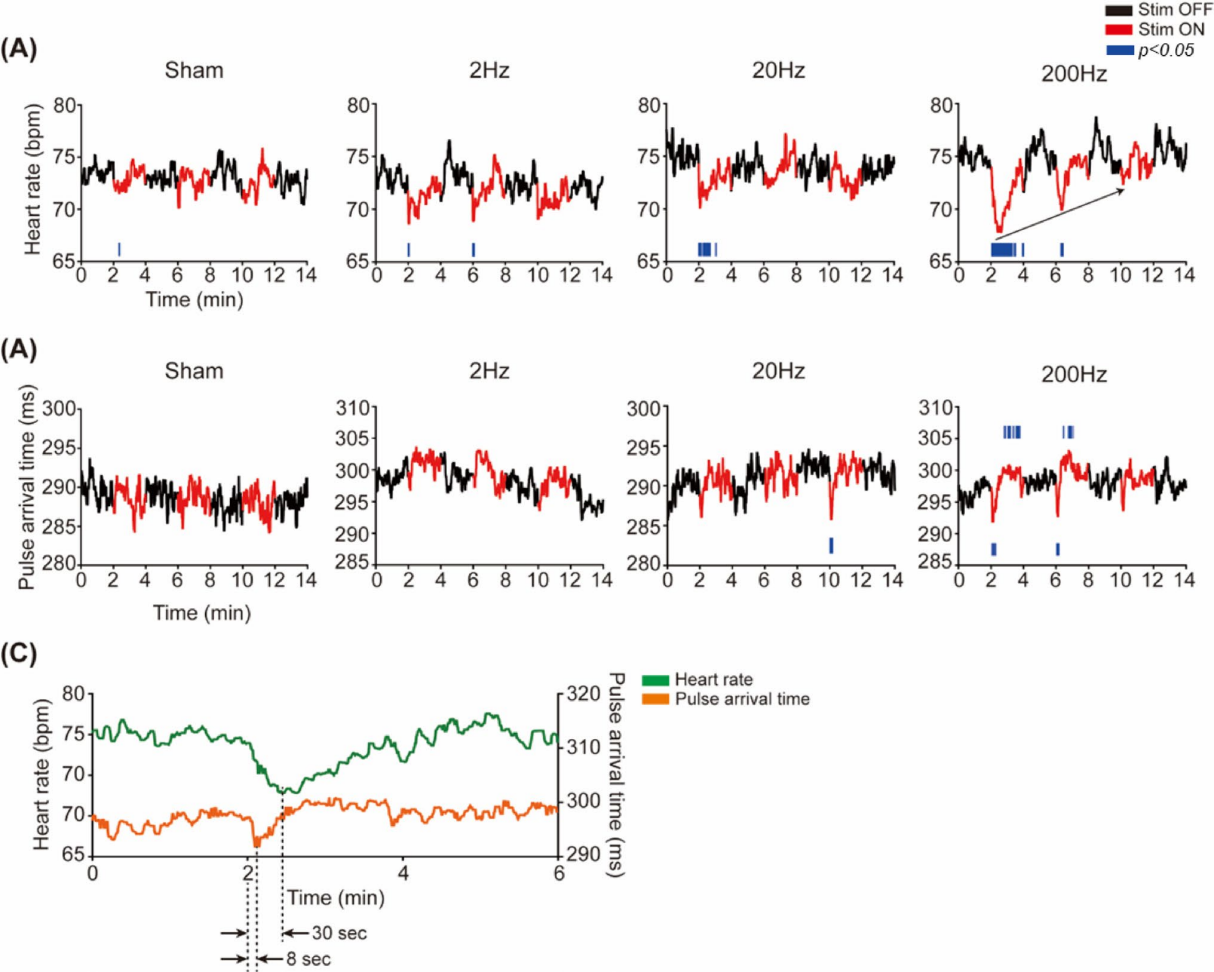
Cardiovascular response according to stimulation frequency group. **A** HR and **B** PAT temporal changes during TN stimulation across different frequency groups. The black and red lines represent the mean HR and PAT during the stimulation OFF and ON periods, respectively. Statistically significant time points compared to the preceding stimulation OFF period are highlighted with blue lines (*p* < 0.05, two-tailed test with Bonferroni correction for multiple comparisons): lines plotted above the trace indicate significant increases, whereas lines plotted below indicate significant decreases. **C** Representative HR and PAT changes during the first 6 min (stimulation from 2 to 4 min) in the 200 Hz stimulation group, with green and orange lines indicating the mean HR and PAT, respectively

When TN stimulation was applied three times at 2-min intervals, unlike the sham group that received no stimulation, all stimulation groups exhibited rapid transient decreases in HR immediately following stimulation onset, demonstrating frequency-dependent responses (Fig. 3A). In the 2 Hz group, the first stimulation induced a maximum 6.56% reduction from the preceding OFF period average (73.12 ± 11.76 bpm) to 68.62 bpm, followed by rapid recovery. The 20 Hz group showed a similar pattern with a maximum 6.8% reduction from the preceding OFF period average (75.23 ± 12.52 bpm) to 70.44 bpm during the first stimulation period. Most remarkably, the 200 Hz group exhibited the most pronounced changes, with a maximum 10.77% reduction from the preceding OFF period average (74.98 ± 7.94 bpm) to 67.69 bpm and demonstrated slower responses for both the decline and recovery compared to other groups.

Notably, repeated stimulation resulted in progressive attenuation of HR responses, indicating a habituation effect that exhibited distinct frequency-dependent patterns. Phase-averaged analysis (Table 2) revealed that the 2 Hz group showed a 1.93 bpm decrease from 73.12 ± 11.76 to 71.19 ± 11.06 bpm during the first stimulation, with decreases of 1.29 bpm during the second stimulation and 1.45 bpm during the third stimulation. In contrast, the 20 Hz group demonstrated a significant reduction only during the first stimulation, decreasing from 75.23 ± 12.52 bpm to 72.65 ± 11.16 bpm by 2.58 bpm (*p* < 0.05), while subsequent stimulations showed no significant responses. The 200 Hz group displayed a more gradual habituation pattern. Following the most substantial 3.90 bpm reduction during the first stimulation (74.98 ± 7.94 → 71.08 ± 8.44 bpm, *p* < 0.05), the second stimulation still produced a significant 1.64 bpm decrease (75.12 ± 8.04 → 73.48 ± 7.67 bpm, *p* < 0.05). In contrast, the third stimulation showed no significant changes (75.44 ± 8.81 vs. 74.29 ± 8.55 bpm, n.s.).

**Table 2:** HR responses during repeated TN stimulation demonstrating habituation effects across stimulation cycles

Group	OFF	ON	*p*	OFF	ON	*p*	OFF	ON	*p*
	0–2 min	2–4 min		4–6 min	6–8 min		8–10 min	10–12 min	
Sham	73.66 ± 10.06	73.05 ± 9.73	n.s	72.98 ± 9.59	72.85 ± 9.73	n.s	73.65 ± 10.15	72.62 ± 9.71	n.s
2 Hz	73.12 ± 11.76	71.19 ± 11.06	*p* < *0.05*	73.39 ± 11.23	72.10 ± 11.52	*p* < *0.05*	72.39 ± 11.75	70.94 ± 11.54	*p* < *0.05*
20 Hz	75.23 ± 12.52	72.65 ± 11.16	*p* < *0.05*	74.11 ± 11.18	73.89 ± 11.12	*n.s*	73.98 ± 10.47	72.84 ± 12.28	*n.s*
200 Hz	74.98 ± 7.94	71.08 ± 8.44	*p* < *0.05*	75.12 ± 8.04	73.48 ± 7.67	*p* < *0.05*	75.44 ± 8.81	74.29 ± 8.55	*n.s*

Mean HR (bpm ± SD) values are shown for each 2-min OFF and ON period pair across three repeated stimulations. Statistical comparisons between consecutive OFF and ON periods were performed using paired two-tailed t-tests, with significant differences indicated by *p* < *0.05*. n.s. = not significant

Meanwhile, PAT analysis revealed that PAT demonstrated a faster response than HR and exhibited rapid transient decreases immediately following stimulation onset (Fig. 3B). No distinct changes were observed in the sham group and 2 Hz group, while the 20 Hz group showed transient decreases with short latency followed by rapid recovery patterns. The 200 Hz group exhibited the most pronounced responses, displaying a characteristic reaction pattern in the first and second stimulation periods where initial transient decreases immediately following stimulation onset were followed by a significant increase above baseline levels. Specifically, during the first stimulation, PAT decreased approximately 1.34% from the preceding OFF period average (296.50 ± 1.30 ms) to a minimum of 292.53 ms, followed by recovery and significant increases. The second stimulation demonstrated a similar pattern to the first stimulation, albeit with attenuated reduction magnitude, decreasing 1.13% from 294.4 ± 12.46 to 290.66 ms. At the same time significant elevation responses above the preceding OFF period persisted. Conversely, the third stimulation period showed loss of these characteristic response patterns with no significant changes observed.

The minimum time for PAT was approximately 8 s, significantly shorter than the 30 s for HR, indicating shorter latency and faster response initiation (Fig. 3C). These characteristic PAT response patterns are presumed to reflect the complexity of peripheral vascular autonomic regulation in response to TN stimulation and represent distinctive physiological characteristics that are distinguishable from HR responses.

### Duration-dependent cardiac adaptation to trigeminal nerve stimulation

**Fig. 4:**
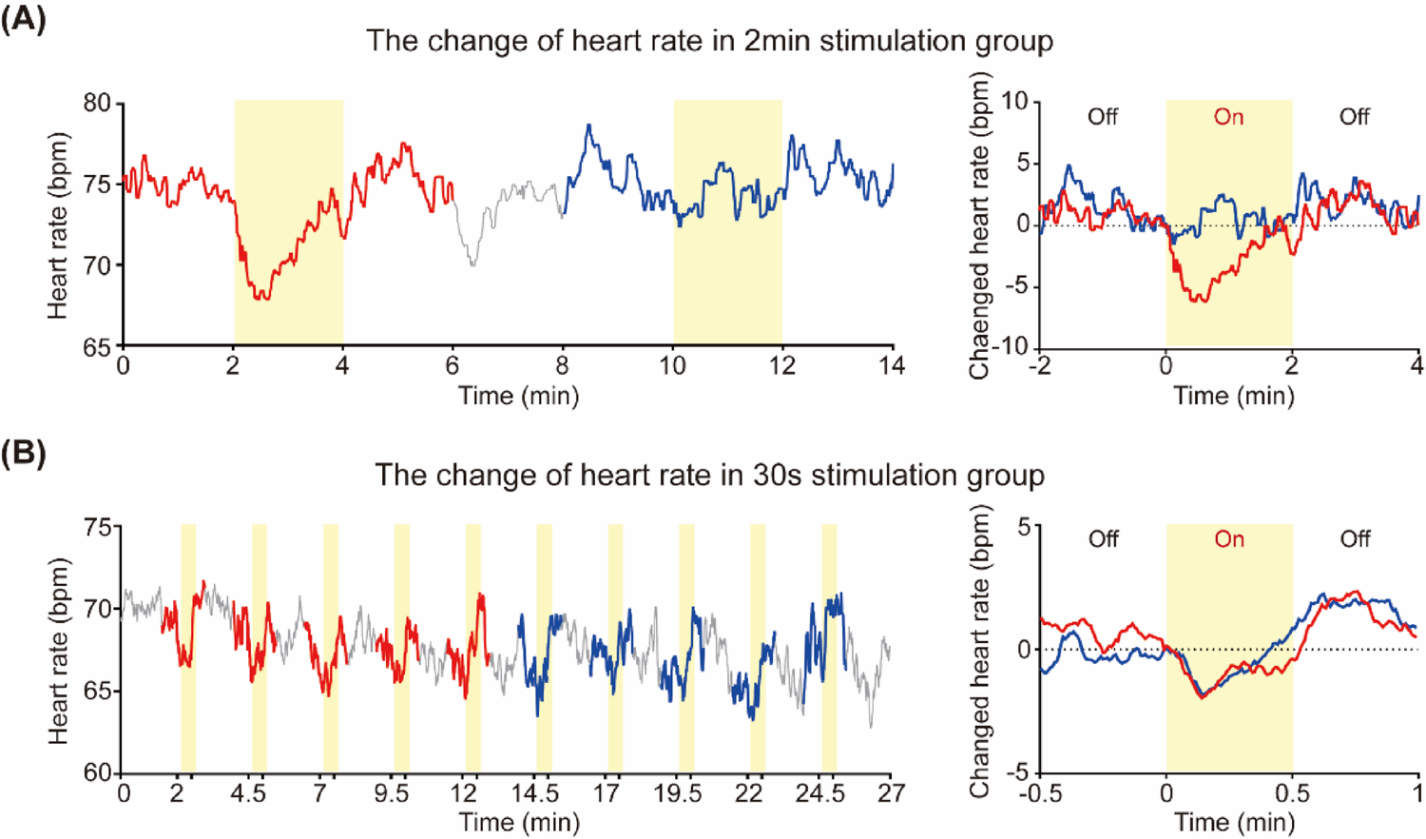
Heart rate response by stimulation duration. **A** Temporal changes in HR according to 2-min duration three consecutive TN stimulation durations. **B** Temporal changes in HR according to 30 s duration stimulation. The red and blue lines represented the HR at the initial and last phases during the stimulation off and on sections, respectively

As shown in Fig. 4A, when 2-min duration stimulation was applied, the first stimulation produced significant changes in HR, while consecutive stimulations led to adaptation, resulting in no HR changes by the third stimulation. In this study, we added an experiment with 10 repetitions of 30-s duration stimulation to examine changes in adaptation according to TN stimulation duration (Fig. 2D). As shown in Fig. 4B, to analyze HR adaptation to repeated stimulation, we compared the average HR response to the first 5 stimulations with the average HR response to the last 5 stimulations. During 30-s TN stimulation, HR decreased and subsequently returned to baseline, similar to the 2-min stimulation pattern. However, when comparing the early and late stimulation periods, no change in this pattern was observed. We confirmed that 30-s stimulation resulted in reduced adaptation response.

### Correlation between resting LF/HF ratio and trigeminal nerve stimulation response

To evaluate the effect of TN stimulation on ANS activity, we assessed the relationship between the LF/HF ratio in the rest phase and the first stimulation ON phase. The changes in the LF/HF ratio within each stimulation group (2 Hz, 20 Hz, and 200 Hz) were not statistically significant. Specifically, the mean LF/HF ratio decreased from 5.76 ± 3.96 at rest to 4.49 ± 3.99 during 2 Hz stimulation, from 4.96 ± 4.61 to 3.56 ± 2.52 during 20 Hz stimulation, and remained relatively unchanged during 200 Hz stimulation (3.67 ± 2.32 at rest vs. 3.66 ± 2.63 during stimulation).

**Fig. 5:**
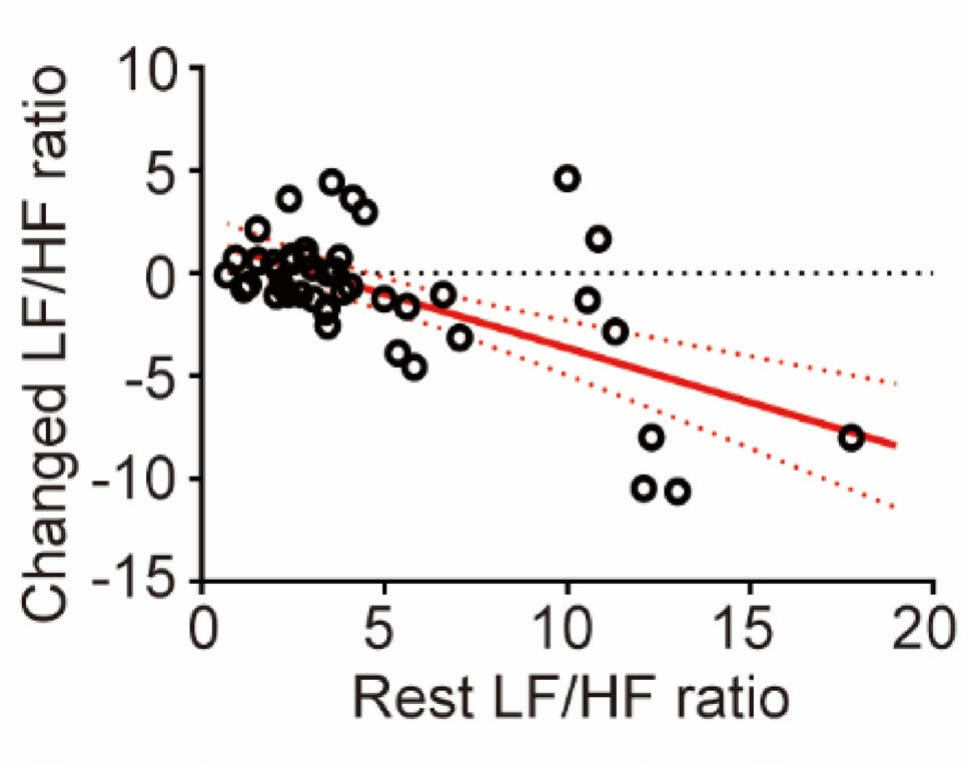
HRV analysis across all TN stimulation groups (2, 20 and 200 Hz; sham excluded; N = 45). The correlation between the LF/HF ratio of in the rest and the change in LF/HF ratio during the first TN stimulation. The black circles represented the data for each subject, and the red solid and dotted lines represented correlations and 95% confidence intervals (Spearman’s r = − 0.4473, 95% CI [− 0.6599, − 0.1683], *p* < 0.05)

Importantly, interindividual analysis showed that subjects with a higher baseline LF/HF ratio (indicating sympathetic predominance) experienced a more pronounced reduction in LF/HF ratio upon TN stimulation, as shown in Fig.5. Correlation analysis confirmed a significant negative relationship between resting LF/HF ratio and the magnitude of change following stimulation (Spearman’s r = − 0.4473, 95% CI [− 0.6599, − 0.1683], *p* < 0.05). This correlation suggests that TN stimulation may modulate autonomic balance more effectively in individuals with elevated sympathetic tone at baseline.

## Discussion

This study investigated the cardiovascular effects of non-invasive TN stimulation across different frequencies in sixty-one healthy adults. Our findings demonstrated that TN stimulation modulates frequency-dependent cardiovascular responses, with higher frequency (particularly 200 Hz) eliciting more pronounced effects on both HR and PAT compared to lower frequencies (2 Hz and 20 Hz). PAT changes preceded HR changes, indicating different temporal dynamics between these cardiovascular parameters as shown in Fig. 3. Habituation effects observed with repeated 2-min stimulations were minimized using 30-s stimulation protocols, suggesting that recovery intervals of at least four times the stimulation duration may prevent adaptation. In the HRV analysis across all stimulation groups, participants with higher baseline LF/HF ratio, indicative of sympathetic hyperactivity, exhibited greater changes in LF/HF ratio in response to TN stimulation. Collectively, our study revealed frequency-dependent cardiovascular effects of TN stimulation, with individual variability based on baseline autonomic status.

These cardiovascular findings complement recent TN stimulation research. Cheng et al. [21] reported that 120 Hz TN stimulation induced increases in mean RRI and decreases in LF/HF ratio, demonstrating the autonomic modulation effects of high-frequency stimulation. It supports our observation of more pronounced effects at higher frequencies. However, prior studies have typically examined single frequency condition, and systematic investigations across various stimulation frequencies in healthy adults remain scarce. To address this gap in the literature, our study systematically examined cardiovascular responses across a broader frequency spectrum, applying TN stimulation at 2, 20, and 200 Hz to comprehensively evaluate frequency-dependent effects. Our data showed similar moderate effects at lower frequencies (2 Hz and 20 Hz), with distinctly larger cardiovascular responses at 200 Hz. While the physiological mechanisms underlying these frequency-dependent patterns are not fully elucidated, it is hypothesized that high-frequency stimulation delivers greater energy, thereby inducing more effective physiological changes. These results indicate that stimulation frequency is an important parameter that should be considered when designing TN stimulation protocols for cardiovascular applications.

The observed PAT patterns, particularly evident in Fig. 3B, provide insights into BP dynamics during TN stimulation. Numerous studies have established an inverse relationship between PAT and BP, validating PAT as a reliable BP estimation parameter [22–26]. The 200 Hz stimulation group demonstrated a pattern suggesting an initial sharp BP increase (PAT decrease) followed by a decrease below baseline values. This temporal sequence likely reflects baroreflex-mediated BP regulation, which modulates cardiovascular function through both peripheral resistance (adrenergic component) and heart rate changes (vagal component) [27]. As illustrated in Fig. 3C, the rapid PAT reduction preceded HR’s decrease during stimulation, suggesting baroreflex engagement in maintaining homeostasis by controlling BP in response to parasympathetic activation induced by TN stimulation.

A distinctive feature of our findings was the habituation pattern observed in HR responses to 200 Hz stimulation (Fig. 4A). Habituation, characterized by diminished responses to repeated stimuli, presents a challenge for sustained therapeutic effects [28]. While some researchers suggest modulating stimulus parameters like frequency or amplitude to mitigate habituation, recent recommendations favor maintaining fixed frequency bands while progressively increasing current intensity to maximum tolerated levels throughout treatment sessions [28]. Our study further explored whether manipulating stimulus timing protocols could reduce habituation effects. Considering that HR decreases in the 200 Hz group reached their nadir at approximately 30 s post-stimulation, we designed a protocol with 30-s stimulation periods separated by 2-min intervals. Testing this protocol on 10 subjects (5 males, 5 females, age: 23.9 ± 1.92) from the 200 Hz group revealed that both HR and PAT responses maintained consistent magnitudes between the first five and last five stimulation periods when the recovery interval was at least four times longer than the stimulation duration (Fig. 4B). These findings suggest an alternative approach to minimizing habituation effects by optimizing the ratio between stimulation and recovery periods, with optimal results requiring recovery intervals at least four times longer than stimulation durations.

Additionally, our HRV analysis revealed a significant negative correlation between resting LF/HF ratio and stimulation-induced changes in this metric, indicating that TN stimulation particularly influences autonomic balance in individuals with sympathetic hyperactivity. This finding suggests potential therapeutic applications for conditions characterized by autonomic dysregulation, where TN stimulation might help restore parasympathetic-sympathetic balance.

However, several limitations of this study should be considered when interpreting the findings. First, because this study enrolled only young and healthy adults, the findings may not be generalizable to other populations or those with varying health conditions. Future studies should include participants across a wider range of populations to more comprehensively assess the physiological effects of TN stimulation. Second, as this was a basic experimental study conducted in healthy subjects, there is a lack of clinical validation for the effectiveness of TN stimulation. Future work should include clinical populations, such as patients with autonomic dysfunction or cardiovascular diseases, to evaluate the therapeutic potential and practical applicability of TN stimulation. Third, the present work focused on acute physiological responses, without assessing the long-term effects or safety of repeated TN stimulation. Follow-up studies with longitudinal designs and repeated stimulation protocols are necessary to evaluate the sustainability and safety of TN stimulation as a therapeutic approach.
